# SBRT: An Opportunity to Improve Quality of Life for Oligometastatic Prostate Cancer

**DOI:** 10.3389/fonc.2015.00101

**Published:** 2015-05-05

**Authors:** Gregory Azzam, Rachelle Lanciano, Steve Arrigo, John Lamond, William Ding, Jun Yang, Alexandra Hanlon, Michael Good, Luther Brady

**Affiliations:** ^1^Department of Radiation Oncology, Drexel University College of Medicine, Philadelphia, PA, USA; ^2^Philadelphia CyberKnife Center, Delaware County Memorial Hospital, Havertown, PA, USA; ^3^Office of Nursing Research, School of Nursing, University of Pennsylvania, Philadelphia, PA, USA

**Keywords:** SBRT, oligometastases, prostate cancer, androgen deprivation therapy, docetaxel

## Abstract

**Objective:**

Oligometastatic prostate cancer is a limited metastatic disease state in which potential long-term control is still possible with the use of targeted therapies such as surgery or stereotactic body radiation therapy (SBRT). SBRT may as well potentially prolong the time before the initiation of androgen deprivation therapy (ADT) and docetaxel chemotherapy for oligometastatic prostate cancer. The goal of this study is to outline prognostic factors associated with improved outcome with SBRT for metastatic prostate cancer and to quantify the effect of prior systemic treatments such as ADT and docetaxel on survival after SBRT.

**Methods:**

Twenty-four prostate cancer patients were treated with SBRT at the Philadelphia CyberKnife Center between August 2007 and April 2014. Retrospective data collection and analysis were performed for these patients on this Institutional Review Board approved study. Kaplan–Meier methodology was utilized to estimate and visually assess overall survival (OS) at the patient level, with comparisons accomplished using the log-rank test. Unadjusted hazard ratios were estimated using Cox proportional hazards regression modeling.

**Results:**

An improved median survival was noted for patients with oligometastatic disease defined as ≤4 lesions with median survival of >3 years compared with 11 months for polymetastases (*p* = 0.02). The use of docetaxel at some time in follow-up either before or after SBRT was associated with decreased survival with median survival of 9 months vs. >3 years (*p* = 0.01).

**Conclusion:**

Prognosis was better for men with recurrent prostate cancer treated with SBRT if they had ≤4 metastases (oligometastases) or if docetaxel was not necessary for salvage treatment. The prolonged median OS for men with oligometastases in this population of heavily pretreated prostate cancer patients following SBRT may allow for improved quality of life because of a delay of more toxic salvage therapies.

## Introduction

According to recent reporting by the National Cancer Institute, ≈15% of men will be diagnosed with prostate cancer in their lifetime (1). In 2011, there were around 2.7 million men living with prostate cancer in the United States alone. If at the time of diagnosis, disease is confined to the prostate gland and surrounding lymph nodes, the 5-year survival rate approaches 100%; but if distant metastases are present, this rate falls to 28% (1). However, metastatic lesions are not all alike. In 1995, Hellman and Weichselbaum first proposed the idea of oligometastatic cancer, an intermediate state on a spectrum between localized and widespread cancer. By definition, oligometastatic cancer is a disease state in which long-term control is still possible (2). The epitomization of this is seen in liver metastasis from primary colon cancers and lung metastasis from sarcoma because resecting these lesions can be curative. Today, oligometastatic cancers are identified as having a unique biological profile, one that limits its metastatic potential. In this context, the use of targeted therapies, such as stereotactic body radiation therapy (SBRT), may serve to control further spread of the disease (3). Efforts have been made to combine SBRT with systemic therapies when there is only a limited extent of metastasis, but the contribution of this strategy to progression free survival or overall survival (OS)is yet to be determined for any particular cancer type (4).

Androgen deprivation therapy (ADT) is the mainstay of treatment for recurrent/metastatic prostate cancer after local therapy, and this therapy is associated with significant decreases in sexual quality of life, increased risk of skeletal fractures, cardiovascular-related mortality, and insulin resistance (5, 6). Efforts have been made to reduce the overall use of ADT, and intermittent ADT have shown similar efficacy for disease control when compared with continuous ADT (7). Recent preliminary data suggests the use of SBRT in salvage therapy for metastatic disease is an effective means for preventing biochemical relapse (8). Bhattasali et al. suggests that castrate-resistant clones are present early in metastatic disease; hence, SBRT therapy for oligometastatic lesions may serve to delay disease progression (9). Berkovic and colleagues’ recent publication suggests that SBRT utilization for prostate oligometastasis delayed the use of palliative ADT by a median of 38 months in a group of 24 patients (10). Decaestecker et al. also recently published similar results (11). Currently, the “Surveillance or metastasis-directed Therapy for OligoMetastatic Prostate cancer recurrence (STOMP),” designed to assess the efficacy of SBRT or surgery for controlling oligometastatic disease (12), is in phase II clinical trials. The primary goal of this trial is to prolong the time before the initiation of palliative ADT, and one endpoint of this study is ADT-free survival.

Docetaxel is the mainstay therapy for castrate-resistant prostate cancer. The S9916 trial reported docetaxel as a second-line agent that could improve OS (13). However, in this trial, the median time to progression in patients receiving docetaxel was only 6.3 months and the OS was 17.5 months (13). The TAX 327 trial reported median survival of patients with castrate-resistant prostate cancer of 19.2 months when treated with docetaxel (14). Currently, a variety of therapies have been implemented in a post-docetaxel setting with modest successes (15). To our knowledge, no publications address the contribution of SBRT to OS after docetaxel therapy. The goal of this study is to outline prognostic factors associated with improved outcome with SBRT for metastatic prostate cancer and to quantify the effect of prior systemic treatments such as ADT and docetaxel on survival after SBRT.

## Materials and Methods

### Patients

Twenty-four prostate cancer patients were treated with SBRT at the Philadelphia CyberKnife Center between August 2007 and April 2014. Retrospective data collection and analysis were performed for these patients. The Institutional Review Board (IRB) of the Crozer Keystone Health System granted approval for this study. Eligibility for inclusion in this study was the previous biopsy-proven diagnosis of prostate cancer and the previous treatment of the disease. Confirmation of prostate metastasis was provided using biopsy (*n* = 8), magnetic resonance imaging (MRI, *n* = 7), positron emission tomography/computed tomography (PET/CT, *n* = 3), or CT alone (*n* = 6). Metastatic workup included the use of a (99m)Tc-methylene (MDP) bone scan, PET/CT, CT, MRI, or both CT and MRI. All patients had progression of prostate cancer documented by rising PSA.

### Treatment

Stereotactic body radiation therapy with 6 mV photons was administered using the CyberKnife system (Accuray Incorporated, Sunnyvale, CA, USA). CT was obtained for treatment planning, which was performed using Multiplan software. Contouring of metastases or adenopathy (CTV) and organs at risk (OAR) in proximity was performed. Dose constraints for normal tissues were previously described by Timmerman and were implemented for OAR (16). The gross target volume (GTV) was equal to the clinical target volume (CTV), and a uniform 5 mm CTV expansion was added for planning target volumes (PTVs). At times, margins were reduced to ≤3 mm when needed for proximal normal tissues. Local failure is defined as recurrence within the CTV. Dose was prescribed to the 60–80% isodose line to cover 95% of the PTVs with the prescribed dose. Tracking was performed using 6D Skull or Xsight Spine or with fiducial markers if necessary, and synchrony tracking was performed as warranted by the treatment site on a case-by-case basis. Treatment delivery was accomplished with between 80 and 150 beams and tracking images were taken every three beams.

### Statistics

Descriptive statistics were used to describe the study population. Recurrence patterns are recorded after first course of SBRT. Kaplan–Meier methodology was utilized to estimate and visually assess OS at the patient level from first course of SBRT for oligometastases. The log-rank statistic was used to compare survival profiles by ADT and docetaxel treatments, in addition to the following measures: age dichotomized at 65 years, PSA decline after SBRT, CTV volume (cut at median CTV for all metastasis), Gleason score, lymph node or other site metastasis, and oligometastatic (≤4 lesions) vs. polymetastatic disease. Cox proportional hazards modeling was used to estimate unadjusted hazard ratios (HRs). As power was limited because of a small sample size, adjusted multivariable Cox proportional hazard models were not estimated. For all unadjusted models, a *p*-value of <0.05 was considered as statistically significant.

## Results

### Patients

The median age of patients at the time of SBRT therapy was 69 years (53–88). A majority of our patients had Gleason scores of ≥8 at the time of diagnosis (*n* = 13). The majority of our patients were initially treated with intensity-modulated radiation therapy (IMRT) for prostate cancer at the time of diagnosis (*n* = 11). Five patients underwent a prostatectomy, four patients were treated with hormone and chemotherapy, two patients received brachytherapy seed implants, one received SBRT, and another cryotherapy. All patients had previously received ADT as part of the initial treatment regimen, except one patient who had undergone prostatectomy. Nine of our patients had oligometastatic disease, defined as having four or fewer lesions. Nearly all of the patients were considered to have castrate-resistant cancer at the time of SBRT (*n* = 20), and 15 patients had also progressed after receiving docetaxel therapy. SBRT dose was based on lesion size and location with the median dose of 24 Gy (18–50) received in three to five fractions. The sites of treatment included bone and lymph node in the majority of patients. Five patients received more than one course of SBRT after at least 1 month from the initial treatment start date. In total 39 sites were treated with SBRT in this patient cohort. The median CTV was 21.9 cm^3^. Less than half of the sites that received SBRT had previous external beam radiation to the SBRT site. A summary of all patient and treatment baseline characteristics at diagnosis, at SBRT, and after SBRT is given in Table 1. The vast majority of patients had no adverse reaction to the treatment. One patient experienced grade 1 diarrhea and another patient reported grade 2 pelvic pain.

**Table 1:** **Patient characteristics**.

Characteristic value	Number
**At primary diagnosis**
Age	
Median	62
Range	52–80
Serum PSA (ng/mL)	
Median	13
Range	1–181
Gleason score	
Median	8
Range	6–10
Treatment modality, *n* (%)	
Primary IMRT	11 (45.8)
Primary prostatectomy	5 (20.8)
Hormone and chemotherapy	4 (16.7)
Seed brachytherapy	2 (8.3)
Cryotherapy	1 (4.2)
SBRT	1 (4.2)
Time initial diagnosis to SBRT (mo)	
Median	51
Range	2–229
ADT initial treatment, *n* (%)	23 (95.8)
**At SBRT**
PSA (ng/mL)	
Median	9
Range	0–1806
Age (years)	
Median	69
Range	53–88
Location of lesions, *n* (%)	
Bone	15 (62.5)
Lymph node	7 (29.2)
CNS	1 (4.2)
Lung	1 (4.2)
Number of metastasis, *n* (%)	
≤4	9 (37.5)
>4	15 (62.5)
CTV (cm^3^)	
Median	21.9
Range	0.6–626.8
Previous radiation to SBRT site, *n* (%)	10 (41.7)
Systemic treatment, *n* (%)	
None or not ADT refractory	4 (16.7)
ADT refractory	5 (20.8)
ADT + docetaxel received	15 (62.5)
**After SBRT**
PSA (ng/mL)	
Median	6
Range	0–554
Recurrence, *n* (%)	
None	11 (45.8)
Local (in SBRT field)	1 (4.2)
Distant (out of SBRT field)	12 (50.0)

### Survival

Gleason score, CTV, previous radiation to CTV, decrease in PSA, and age were not associated with OS after SBRT in our analysis on the basis of the log-rank statistic and Kaplan Meier estimates (*p* = 0.76, 0.36, 0.28, 0.29, and 0.25, respectively). Although not statistically significant, there was a trend for enhanced survival in patients that had metastases in lymph node sites vs. any other site (*p* = 0.15, data not shown). A decrease in PSA after SBRT was not a prognostic indicator for OS. Of the 15 patients who had follow-up PSA after SBRT, nine had decrease in PSA. PSA, however, was useful to track progression of disease and guided further metastatic workup.

**Figure 1:**
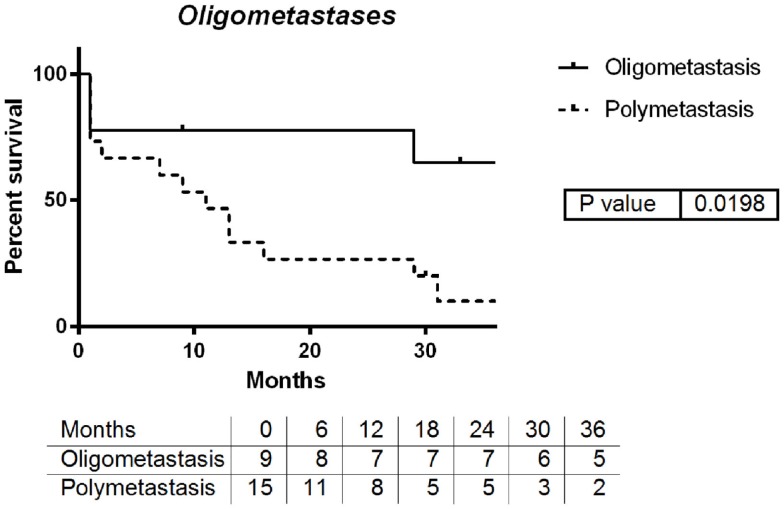
**Survival by number of metastatic lesions: oligometastases vs. polymetastases**.

An improved median survival was noted for patients with oligometastatic disease with median survival >3 years compared with 11 months for polymetastases (log-rank *p* = 0.0198, Figure 1).

**Figure 2:**
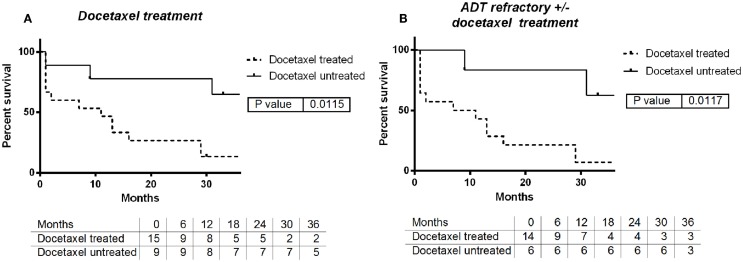
**(A)** Survival by use of docetaxel chemotherapy: yes vs. no. **(B)** Survival by use of docetaxel with ADT refractory: yes vs. no.

The use of docetaxel at some time in follow-up either before or after SBRT was associated with decrease in median survival of 9 months when docetaxel was used vs. >3 years with no use (log-rank *p* = 0.0115, Figure 2A). This effect persists when evaluating only patients with castrate-resistant disease with median survivals of 9 months vs. >3 years (log-rank *p* = 0.0117, Figure 2B). In contrast, there was no significant survival difference between patients that received ADT when compared with those who did not (log-rank *p* = 0.936).

**Figure 3:**
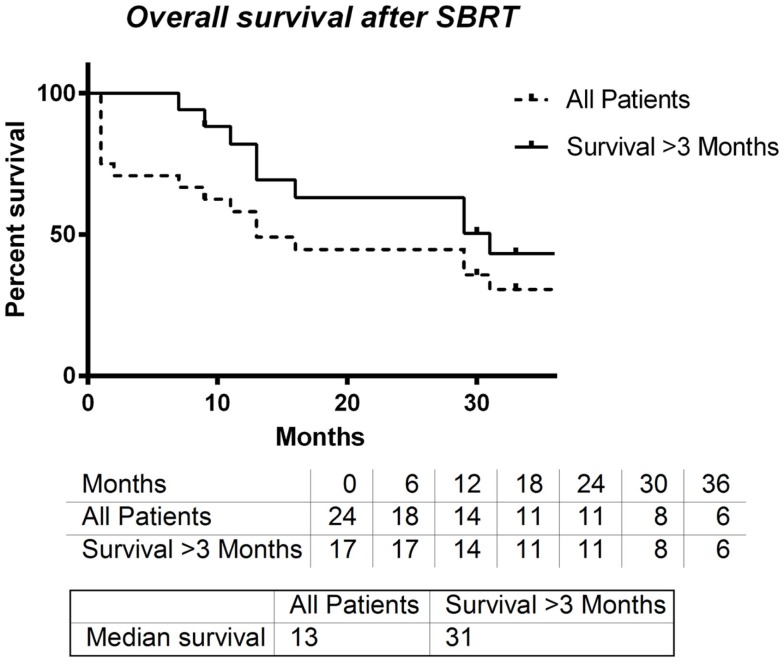
**Overall survival entire group vs. overall survival for patient’s surviving at least 3 months**.

Overall survival after SBRT in all patients was assessed, and the median time until death was 13 months. A small subset of patients died within 3 months after receiving SBRT. When these patients were excluded from this analysis, the median survival time after SBRT was 31 months (Figure 3).

Tables 2 and 3 provides results for univariate Cox proportional hazard regression modeling for all patients and those surviving >3 months after SBRT to reduce bias for patients who were at end of life and treated palliatively. The hazard of death is significantly increased among those with more than four metastatic lesions (HR 3.33, *p* = 0.057; HR 6.52, *p* = 0.048) and treatment with docetaxel (HR 4.16 *p* = 0.027; HR 4.34 *p* = 0.069).

**Table 2:** **Univariate Cox regression model results for all patients**.

	Hazard ratio	Lower CL	Upper CL	Prob ChiSq
ADT + docetaxel received	4.891	0.803	29.785	0.0851
ADT refractory, no docetaxel	1.038	0.123	8.781	0.9726
PSA increased	1.85	0.412	8.305	0.4221
Previous RT in SBRT field	1.432	0.53	3.873	0.479
Age <65 years	1.764	0.657	4.738	0.2599
CTV > 22	1.578	0.573	4.349	0.3778
Gleason score > 7	1.197	0.368	3.898	0.7648
ADT untreated	2.84	0.498	16.19	0.24
Non-lymph node site	3.009	0.869	10.412	0.082
Polymetastatic disease	3.328	0.966	11.473	0.0568
Docetaxel received	4.162	1.174	14.751	0.0272

**Table 3:** **Univariate Cox regression model results for patients surviving >3 months after SBRT**.

	Hazard ratio	Lower CL	Upper CL	Prob ChiSq
ADT + docetaxel received	11.508	0.501	264.091	0.1265
ADT refractory, no docetaxel	3.239	0.119	88.286	0.4859
PSA increased	1.982	0.191	20.608	0.567
Previous RT in SBRT field	3.308	0.826	13.245	0.0909
Age <65 years	2.254	0.596	8.528	0.2312
CTV >22	3.15	0.69	14.374	0.1384
Gleason score >7	1.341	0.261	6.897	0.7257
ADT untreated	6.467	0.318	131.363	0.2243
Non-lymph node site	5.975	0.925	38.582	0.0603
Polymetastatic disease	6.519	1.016	41.816	0.048
Docetaxel received	4.338	0.889	21.164	0.0696

At the conclusion of our study, we had nine patients who never received docetaxel with a median survival time from first treatment with SBRT of 41 months (11–70). We had three patients who did not receive palliative ADT 32, 40, and 70 months following first SBRT treatment. Overall 25% of patients remain free of disease at last follow-up.

## Discussion

In this retrospective study, we report our experience in men with metastatic prostate cancer who were treated with SBRT. Our findings support previous descriptions of the oligometastatic state of prostate cancer. In our experience, those patients with oligometastatic disease treated with SBRT had significantly longer OS times than those that had more than four lesions. This supports other work that suggests that for patients with four or fewer metastatic lesions, targeted therapy, such as SBRT, is an effective means to control disease. Currently, the NRG-BR001 trial aims to more clearly define the dose parameters and side effects of SBRT for oligometastatic disease and includes men with a prostate cancer primary.

Surprisingly, higher Gleason score was not associated with worse survival after SBRT. Recently published data by Rusthoven et al. demonstrated that higher Gleason scores are a strong predictor of decreased OS in patients with metastatic prostate cancer (17). One possible explanation for this discrepancy is that our group was heavily pretreated and not only castrate resistant but also docetaxel resistant which may lead to a more homogeneous high risk population at the time of SBRT with little prognostic value from the Gleason score at initial diagnosis.

Androgen deprivation therapy and docetaxel treatment are standard systemic treatments for metastatic prostate cancer. In our study, we identified two patients with oligometastatic disease who were treated with SBRT and had not yet received palliative ADT. One such patient was treated with SBRT without ADT or docetaxel on three separate occasions over a period of 3 years with no evidence of disease at last follow-up by diagnostic studies and PSA.

In our experience, those patients treated with docetaxel at any time had decreased survival compared with those who had not received this treatment. When evaluating only patients with castrate-resistant disease, we still found that those who had received docetaxel fared worse than those who had not. Considering docetaxel as a second-line agent, it follows that these patients have more advanced disease, which could account for the diminished survival times. However, recent work by Sweeney et al (18). suggests that upfront docetaxel with ADT enhances OS in patients with visceral metastases and/or four or more bone metastases vs. ADT alone (18). We consider our cohort of patients distinct from those treated upfront with docetaxel and ADT, because our patients only received docetaxel as a palliative measure. More work is needed to determine the effect of SBRT in patients treated upfront with docetaxel and ADT, especially in those patients with oligometastatic visceral metastases. Of note, nine of our patients who were treated with SBRT have yet to require docetaxel as a second-line agent. Such end points may speak to the ability of SBRT to improve quality of life in patients with oligometastatic prostate cancer by promoting a longer interval to salvage systemic therapy especially given the low rates of SBRT-related toxicity reported herein.

In all patients who had received SBRT therapy, median OS was 13 months. Owing to the palliative nature of some SBRT treatments, several of our patients were treated at the end of life. When these patients were removed, the median survival time increased to 31 months, which compares favorably with second-line chemotherapy trials.

## Conclusion

Prognosis was better for men with recurrent prostate cancer treated with SBRT if they had four or less metastases (oligometastases) or if they had not required docetaxel treatment. Use of SBRT for oligometastases is an area of active research to hopefully improve quality of life and survival for men with metastatic prostate cancer.

## Conflict of Interest Statement

Drs. Steve Arrigo, Luther Brady, John Lamond, Rachelle Lanciano, and Jun Yang have ownership in Philadelphia CyberKnife. The remaining authors have no conflicts of interest to declare.
